# Currarino triad

**DOI:** 10.11604/pamj.2022.41.143.33419

**Published:** 2022-02-17

**Authors:** Krishna Prasanth Baalaan, Nishanth Gurunathan

**Affiliations:** 1 Department of Community Medicine, Sree Balaji Medical College and Hospital, Bharath Institute of Higher Education and Research, Chennai, Tamil Nadu, India,; 2 Sree Balaji Dental College and Hospital, Chennai, Tamil Nadu, India

**Keywords:** Anorectal, currarino syndrome, meningocele

## Image in medicine

Currarino triad is an autosomal dominant hereditary condition, characterized by triad of sacral agenesis abnormalities, anorectal malformation and presacral mass consisting of teratoma, anterior sacral meningocele or both. It is caused by mutations in the MNX1 gene. We present a case of a 2-year-old male child, brought by his mother with complaints of abdominal distension, failure to pass urine/faeces for 6 days. On examination, swelling of size 4x3cm was seen in the sacrococcygeal region. Magnetic resonance imaging (MRI) of abdomen and pelvis showed large cystic multi lobulated mass in sacrococcygeal region with dural communication evident of an anterior sacral meningocele. One month later, biopsy was done, which showed malignant mixed germ cell tumor in presacral area. Child underwent sacral partial laminectomy, laminoplasty and transdural ligation in neck of meningocele.

**Figure 1:**
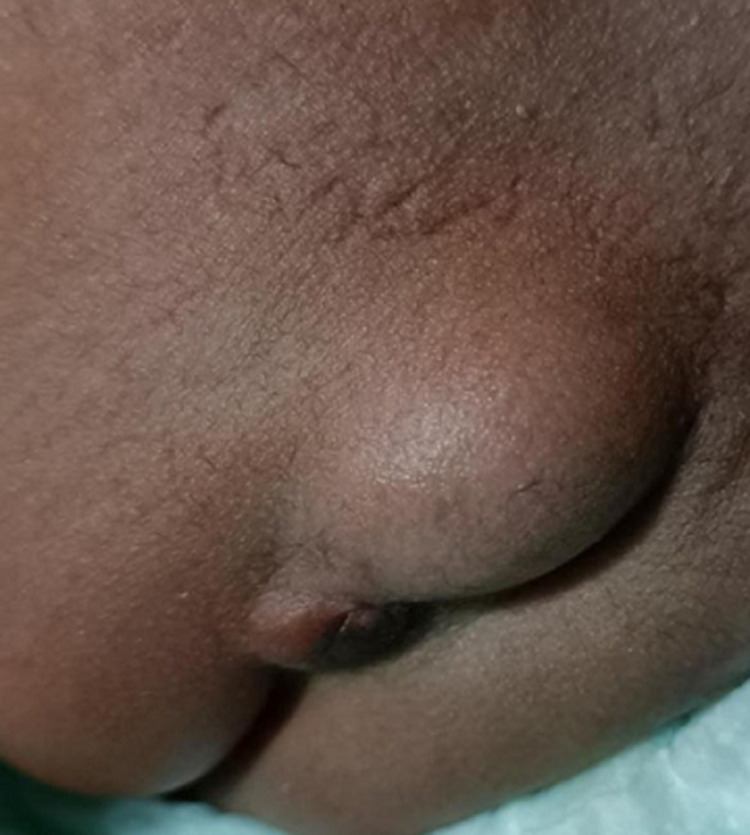
sacrococcygeal swelling 4x3cm in size

